# Interface Characteristics and Bonding Performance of the Corrugated Mg/Al Clad Plate

**DOI:** 10.3390/ma14164412

**Published:** 2021-08-06

**Authors:** Sha Li, Xinyang Liu, Yi Jia, Jianchao Han, Tao Wang

**Affiliations:** 1 College of Mechanical and Vehicle Engineering, Taiyuan University of Technology, Taiyuan 030024, China; tyutls@163.com (S.L.); tyutjiayi@163.com (Y.J.); 2 Engineering Research Center of Advanced Metal Composites Forming Technology and Equipment, Ministry of Education, Taiyuan 030024, China; 3 College of Materials Science and Engineering, Taiyuan University of Technology, Taiyuan 030024, China; liuxinyang20201115@163.com

**Keywords:** Mg/Al clad plate, corrugated roller, interface bonding performance, numerical simulation

## Abstract

In this paper, a corrugated Mg/Al clad plate was successfully manufactured by an upper corrugated roller and a lower flat roller at 400 °C rolling temperature and 35% reduction ratio. Interface bonding performance of the corrugated Mg/Al clad plate was studied by tensile-shear test. The finite element method was used to simulate the corrugated rolling process. Experiment results revealed that the Mg/Al clad plate fabricated by corrugated roller had a tight bonding interface, no crack, and no intermetallic compounds. The transverse tensile-shear strength at the trough position reached 31.22 MPa, and the tensile-shear strength at the peak position was 17.61 MPa. It can be found that the stress and strain of interface metal at the trough position were the largest through numerical simulation results. Two cross-shear zones can be formed in the rolling deformation zone of the corrugated Mg/Al clad plate, which can accelerate the metal plastic flow and promote interface close bonding.

## 1. Introduction

With the rapid development of aerospace, electronic technology, petrochemical, and other emerging industries, it has been difficult to fulfill the comprehensive performance requirements of traditional single-metal material. Therefore, layered metal clad plates have attracted more and more attention [1,2,3]. A layered metal clad plate is a new material that can combine two or more metal plates through a specific preparation process. This kind of metal material has become a hot topic in recent years because of its simple preparation method, low cost, and high bonding strength [4,5].

Metal Mg materials with lightweight, high specific strength, and high specific stiffness are widely used in aerospace, mechanical power, and other fields [6,7,8]. However, low corrosion potential, poor plastic deformability at room temperature, and other factors lead to the high production cost of Mg, which greatly limit the wide use of Mg and its alloys [9,10,11]. Metal Al alloy is a light and environmentally friendly material with active chemical properties, which can easily react to oxygen in the air to form a dense oxide film. Al alloy has good corrosion resistance and plastic deformation ability, which is suitable for various forming methods [12,13]. Al and its alloys are widely used in transportation, marine vessels, artificial satellites, and other fields. Therefore, combining metal Mg and metal Al to make a Mg/Al clad plate can give full play to the advantages of basic materials. A Mg/Al clad plate has a smaller density, higher strength, and good corrosion resistance [14,15].

At present, there are many methods to prepare Mg/Al clad plates through the roller, such as hot-roll bonding, explosive + rolling, accumulative rolling, etc. Luo [16] realized the bonding of Al/Mg/Al three-layer clad plates by the two-pass hot-rolling method at 400 °C. The results indicated that there was no intermetallic compound formed at the interface after the first rolling pass, but the intermetallic compound distributed at the interface in a discontinuous state after the second rolling pass. The author thought that the discontinuous intermetallic compound can limit crack propagation and consume the energy needed for crack propagation, improving the bonding strength of the clad plates. Chen [17] obtained the Mg/Al clad plate through 5-pass hot-rolling after explosive welding, and the rolled Mg/Al clad plates were annealed at different temperatures and holding times. The results revealed that the thickness of interface diffusion increased with annealing temperature and time, and the mechanical properties first increased and then decreased. Wu [18] prepared the Mg/Al clad plate by accumulative rolling at 400 °C. It was found that the tensile strength of the clad plate in the rolling direction and transverse direction decreased during the third cycle, which was due to the necking and cracking of the Al alloy plate.

In recent years, a new corrugated + flat rolling (CFR) process has been developed to prepare Mg/Al clad plates. Some researchers have studied the Mg/Al clad plate through the CFR technique. Wang [19] successfully prepared the Mg/Al clad plate with a corrugated interface and flat surface by the CFR process, and the interface microstructure and mechanical properties were researched. Li [20] also produced the Mg/Al clad plate by the CFR method. The result showed that there was no delamination at the interface and no crack at the base metals after the bending test. Wang [21] researched the interfacial microstructure of the Mg/Al clad plate obtained only by the corrugated roller through the EBSD technology. The above-mentioned studies mainly analyze the CFRed Mg/Al clad plate by experimental means, but few studies are carried out by numerical simulation. By simulating the rolling process of Mg/Al clad plate with a corrugated roller, the parameters can be obtained, such as the change of stress and strain on the plate during the rolling process. Therefore, in this study, numerical simulation and experimental study are combined to study the interface characteristics and bonding performance of the corrugated Mg/Al clad plate, and the relationship between stress and strain on the plate and the interface bonding performance is established.

## 2. Experimental Method and Numerical Simulation

### 2.1. Experimental Method

The original materials used in this study are AZ31B Mg alloy plate and 5052 Al alloy plate. Mg plate and Al plate have the same size with length of 100 mm, width of 60 mm, and thickness of 2 mm. The chemical composition and mechanical properties of the Mg plate and the Al plate used in the experiment are shown in Table 1 and Table 2, respectively.

Before rolling, the metal surfaces to be bonded were ground with a wire brush until fresh metal was exposed. Then the polished surfaces were cleaned with alcohol to remove impurities such as oil stains. Finally, the polished plates were stacked to make the Mg/Al plate blank. The prepared Mg/Al plate blank was put into a tubular heating furnace with a preset temperature and argon protection for heat preservation treatment. The holding temperature was 400 °C and the holding time was 15 min. In this rolling process, a rolling mill with upper corrugated roller and lower flat roller was used. The reduction ratio was set at 35%. In the corrugated rolling process, the Mg alloy plate was in contact with the upper corrugated roller and the Al alloy plate was in contact with the lower flat roller. The experimental roller diameter is 150 mm, and the surface curve of the corrugated roller is sinusoidal. After the rolling experiment, the corrugated Mg/Al clad plate with corrugated Mg plate surface and microwave interface was obtained.

The interface morphology of the corrugated Mg/Al clad plate was observed by scanning electron microscope (SEM, IT500) equipped with Energy Dispersive Spectrometer (EDS). Interfacial bonding strength of the corrugated Mg/Al clad plate was tested by tensile-shear tests, and the sample was cut along the transverse direction (TD). The testing speed was 0.2 mm/min. After the mechanical tests, the fracture surfaces were observed and analyzed by SEM and EDS techniques. The surface roughness of interface metal after rolling was measured by three-dimensional profilometer.

### 2.2. Numerical Simulation

**Figure 1:**
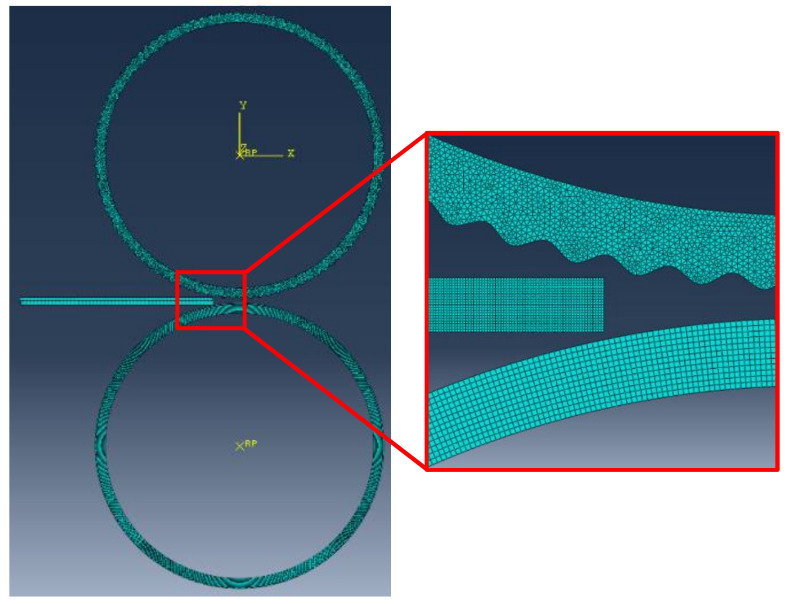
The meshing of the corrugated rolling process.

Using commercial finite element software ABAQUS 6.14-1 and temperature-displacement coupling dynamic explicit analysis method, the elastic–plastic solution of corrugated rolling process is realized. In this study, a two-dimensional rolling model is established. The initial temperature of the metal plate blank is set to 400 °C and the roller temperature is 20 °C. The initial thickness of the Mg plate and Al plate is 2 mm. The numerical simulation process of rolling Mg/Al clad plate with corrugated roller involves the workpiece’s large deformation process, which requires strict mesh division. The calculation amount is too large if the divided mesh is too small, and the calculation accuracy is not high if the divided mesh is too large. Therefore, ALE (Arbitral Lagrange–Euler) method will be used to solve this problem. The mesh generation is shown in Figure 1. To improve the calculation accuracy, plane strain four-node element CPE4RT is selected during the simulation process [22]. In addition, the reduction ratio is 35%, which is the same as the actual rolling parameter.

**Figure 2:**
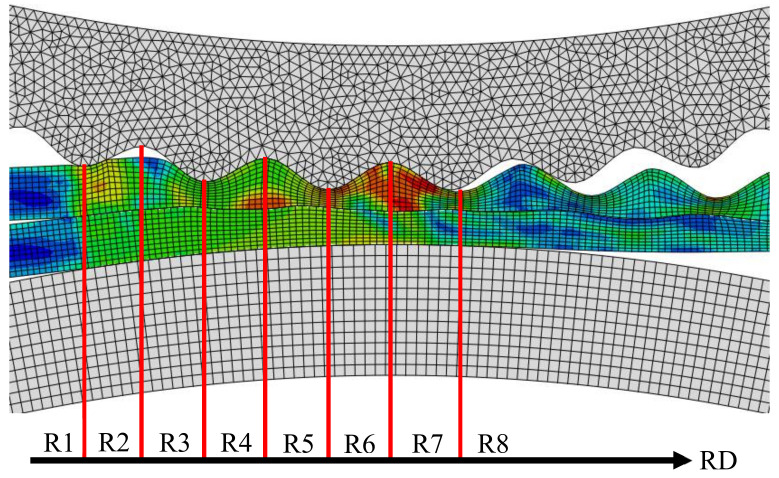
Zoning diagram of the corrugated rolling deformation zone.

The rolling deformation zone of the corrugated Mg/Al clad plate is divided into several small regions for subsequent analysis convenience, as shown in Figure 2. The deformation zone is divided into eight regions, i.e., Region 1 (R1), Region 2 (R2), etc. to Region 8 (R8), from the time when the corrugated roller just contacts with the upper Mg plate to the time when the rolled piece leaves the roller.

## 3. Results and Discussion

### 3.1. Interface Morphology of the Corrugated Mg/Al Clad Plate

**Figure 3:**
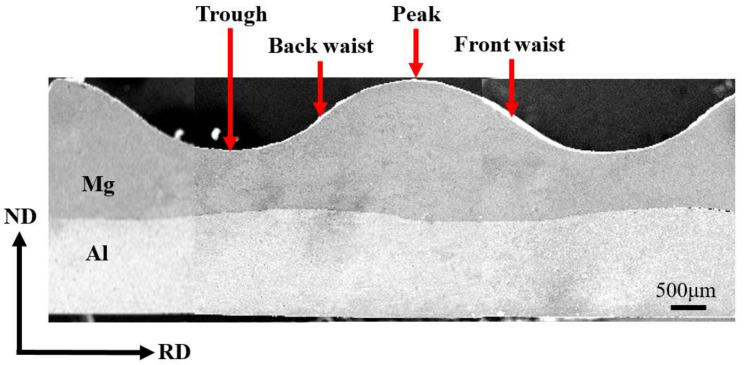
Interface macro-morphology and typical positions of the corrugated Mg/Al clad plate.

The corrugated Mg/Al clad plate is prepared by the rolling mill with an upper corrugated roller and a lower flat roller under conditions of 400 °C temperature and 35% reduction ratio. Figure 3 displays the interface macro-morphology of the corrugated Mg/Al clad plate. To facilitate follow-up research, four positions (front waist, peak, back waist, and trough) on the corrugated plate are defined according to the rolling direction and the Mg surface shape. The area where the metal Mg protrudes upward is called the peak position, and the area where the metal Mg sinks downward is called the trough position. The area before the peak position is called the front waist and the area after the peak position is called the back waist along the rolling direction. The specific positions are shown in Figure 3.

**Figure 4:**
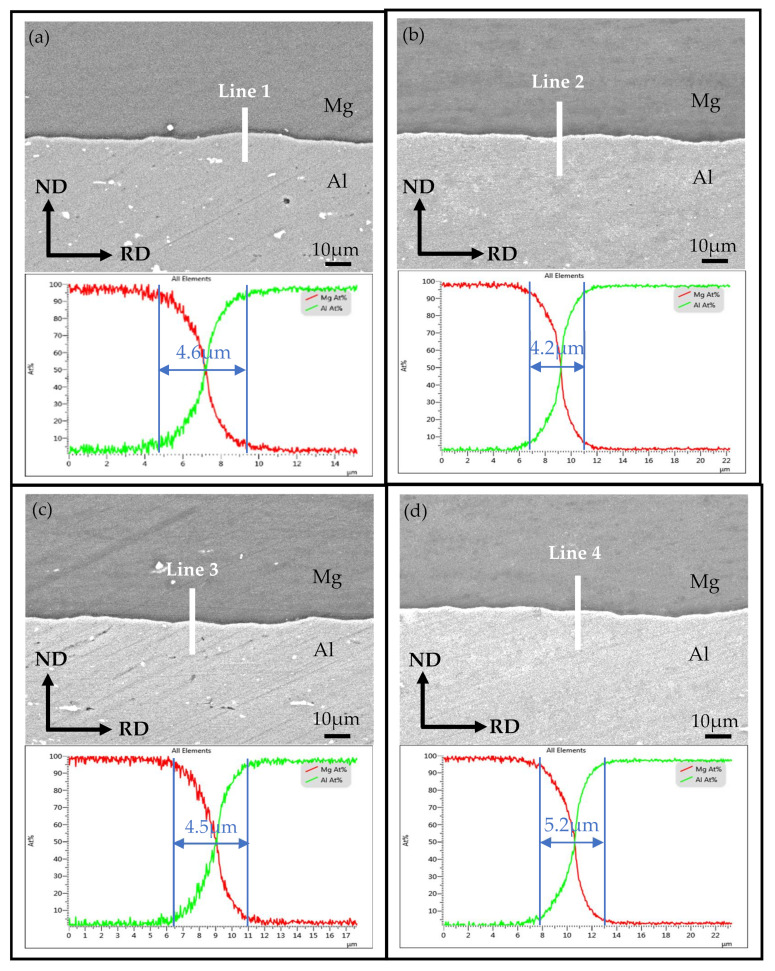
Interface SEM images and corresponding line-scan results of the corrugated Mg/Al clad plate: (**a**) front waist, (**b**) peak, (**c**) back waist, (**d**) trough.

Figure 4 shows the interface SEM images for the typical positions of the corrugated Mg/Al clad plate and the corresponding line-scanning results. From Figure 4, it can be seen that the interfaces of the corrugated plate at the front waist, peak, back waist, and trough positions are well bonded, and there are no holes, cracks, and other defects at the interfaces. Meanwhile, the distribution of Mg element and Al element at the interface has a sudden drop according to the corresponding line-scanning results. The changing trend of elements shows X shape, which indicates that there is no stable hard brittle intermetallic compound layer at the interface. Although a stable intermetallic compound layer is not formed at the interface, the diffusion layer can be observed. It can also be seen from the line-scan diagram shown in Figure 4 that the thickness of the interface diffusion layer at four positions of corrugated Mg/Al clad plate is different. The minimum thickness of the interface diffusion layer at the peak position is 4.2 μm, and the maximum diffusion layer thickness is 5.2 μm at the trough position. The thickness of the interface diffusion layer at the front waist and the back waist positions is between the peak and trough positions. The interface diffusion layer thickness is different at these four positions, but the difference is not obvious.

The surface roughness values of interfacial metal Mg and metal Al at four special positions after corrugated rolling are measured. Table 3 shows the metal surface roughness values. It can be seen from Table 3 that the roughness change trend is consistent with the thickness of the interface diffusion layer of the corrugated Mg/Al clad plate. The larger the roughness value, the thicker the interface diffusion layer. Moreover, the larger rolling force can accelerate the surface fracture of the metal to be bonded and increase the metal roughness. The metal roughness value at the trough position (Mg of 3.597 μm, Al of 4.430 μm) is the highest, which can promote the interface element diffusion and improve the interface bonding quality of the clad plate.

### 3.2. Effect of Stress and Strain on Interface Metal Deformation

**Figure 5:**
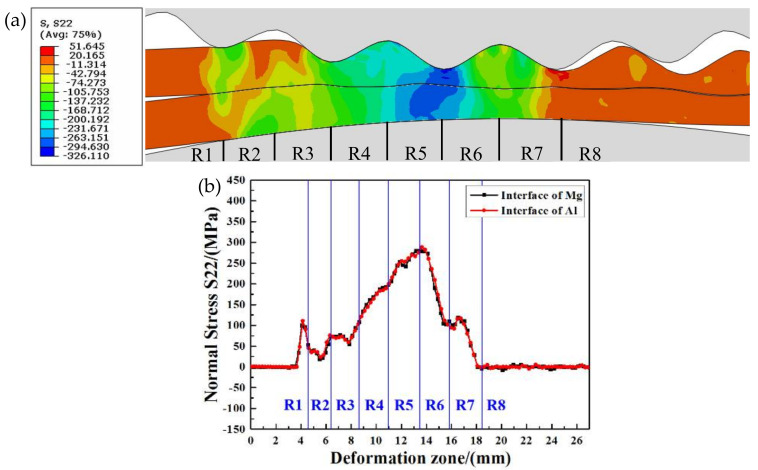
Normal stress distribution of interface metals in corrugated rolling deformation zone: (**a**) normal stress nephogram, (**b**) normal stress curves.

Figure 5 shows the normal stress distribution of the interface metals in the corrugated rolling deformation zone. Figure 5a is the normal stress nephogram, and Figure 5b is the normal stress curve. It can be seen from Figure 5b that the normal stress change trend of metal Mg and metal Al at the bonding interface is highly consistent, and the normal stress curves are almost coincident.

In the R1 region, the upper corrugated roller and the lower flat roller are in primary contact with metal Mg and metal Al, respectively. Under the impact of the roller, the surfaces of Mg and Al are subjected to normal stress, and then the normal stress is transferred to the interface. At this time, the interface metals are suddenly subjected to the external force, and the normal stress increases and shows a linear growth trend. In the R2 region, the normal stress of Mg and Al decreases because the upper corrugated roller does not completely contact with the upper Mg plate. However, the normal stress of the interface metals in this R2 region is not completely reduced to zero, which is caused by the extrusion and pushing action of the roller in the R1 region to the metal in the R2 region. In addition, the normal stress of the metal near the R3 region rises slightly. The normal stress of interface metal fluctuates in the R3 region, and begins to rise near the R4 region until the normal stress in the R4 region continues to rise steadily. This is mainly attributed to the fact that the roller has been in complete contact with the metals since the R4 region, and the normal stress at the interface metal gradually increases as the rolling process continues. The normal stress at the interface metals reaches its peak at the junction of the R5 region and R6 region (279.6 MPa for Mg plate and 288.5 MPa for Al plate). The rolled piece leaves the roller with the rolling process, and the normal stress begins to decrease until it drops to zero.

From Figure 5a, the junction of the R5 region and R6 region is the metal trough position, where the normal stress of the interface metal reaches the maximum value in the whole rolling deformation zone. It can be seen from Figure 5b that the R5 region contains metal at the front waist of the plate, and the normal stress values of interface metal Mg and metal Al at the front waist are 245 MPa and 253.6 MPa, respectively. The normal stress value is higher than that of metal Mg and metal Al at the back waist in the R6 region. The junction of the R6 region and R7 region is the metal peak position, where the normal stress of Mg and Al at the interface is 109.7 MPa and 101.8 MPa, respectively. This normal stress value is the lowest among these four positions of the corrugated Mg/Al clad plate.

**Figure 6:**
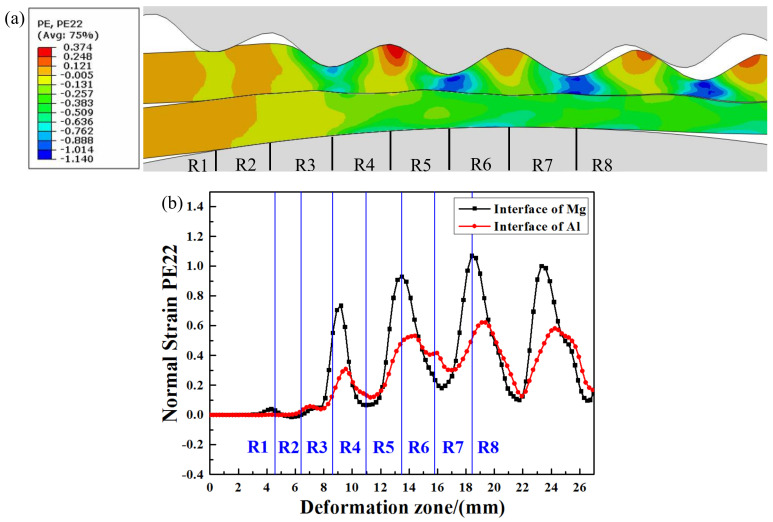
Normal strain distribution of interface metals in corrugated rolling deformation zone: (**a**) normal strain nephogram, (**b**) normal strain curves.

Figure 6 shows the normal strain distribution of the interface metals in the corrugated rolling deformation zone. Figure 6a is the normal strain nephogram and Figure 6b is the normal strain change curve. It can be seen from Figure 6b that the shape of the normal strain curve of interface metals is corrugation, and the amplitude of metal Mg is larger than that of metal Al.

In the R1 region, the normal strain of metal Mg changes slightly, while the normal strain of metal Al is zero. The reason for this is that the Mg plate undergoes plastic deformation and transfer to the interface when the upper corrugated roller contacts with the metal Mg, but there is no plastic deformation in metal Al at this time. In the R2 region, the normal strain of interfacial metals Mg and Al fluctuates, which is caused by the extrusion of the roller in the R1 region. At the junction of the R2 region and R3 region, the interfacial metal normal strain begins to rise synchronously and increase significantly in the R3 region. This is because the deformation occurs after the contact between the rollers and the plates, and the normal strain increases rapidly. In the R4 region, both Mg plate and Al plate reach the normal strain peak values (0.735 for Mg and 0.309 for Al). Because the metals have not entered the stable rolling stage, the peak value of the normal strain of the interface metal does not appear at the junction of the R3 region and R4 region. As the rolling process continues, the upper corrugated roller contacts with the Mg plate at the back waist position, and the deformation of the Mg plate and Al plate decreases relative to the trough position. The normal strain of Mg and Al decreases until the peak position at the junction of R4 and R5. The interface normal strain of the Mg plate rises to the highest point (trough position) at the junction of the R5 region and R6 region (0.93 of Mg plate). However, the Al normal strain reaches the maximum value of 0.51 in the R6 region. This cycle is repeated until the Mg/Al clad plate leaves the roller.

Although the changing trend of normal strain at the interface Mg and Al is consistent, the maximum value of the Mg normal strain is significantly higher than that of the Al plate. The peak value of the Mg normal strain is earlier than that of the Al plate. During the corrugated rolling process, the metal at different positions is subjected to uneven rolling force because of the special shape of the upper corrugated roller, resulting in various plastic deformation degrees of metal at different positions. The plastic deformation of metal Mg in direct contact with the corrugated roller is the largest at the trough position. However, because the deformation resistance of metal Mg and metal Al is different, and the plastic deformation degree of surface metal is greater than that of interface metal, the plastic deformation of metal Mg at the trough position does not penetrate metal Al. Therefore, the peak point of the metal Mg normal strain is higher than that of metal Al.

### 3.3. Research of the Cross-Shear Zone

**Figure 7:**
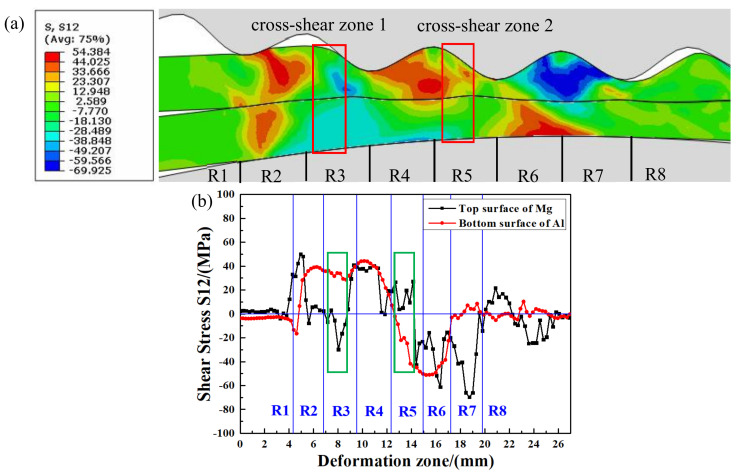
Shear stress nephogram of corrugated rolling deformation zone: (**a**) shear stress nephogram, (**b**) shear stress curves of the sheet surface.

Figure 7 shows the shear stress distribution of the Mg upper surface and the Al lower surface in the corrugated rolling deformation zone. Figure 7a is the shear stress variation nephogram and Figure 7b is the shear stress variation curve. As can be seen from Figure 7b, the magnitude and direction of shear stress on the Mg upper surface change dramatically because of the direct action of the corrugated roller on the Mg plate.

In the R1 region, the shear stress on the Mg upper surface suddenly increases. When the outward protruding part of the corrugated roller is in contact with the Mg plate, it will have an impact on the plate, resulting in a sudden increase in the shear stress of the Mg plate. The first shear stress peak value on the Mg upper surface appears in region R2 due to the rubbing action of the roller, and the shear stress reaches 50.1 MPa. Because the Mg plate and the upper corrugated roller do not completely contact in the R2 region, the shear stress on the Mg upper surface drops to zero and then fluctuates slightly around zero. In the R3 region, the direction of shear stress on the Mg upper surface changes, which is opposite to the direction of shear stress on the Al lower surface. At this time, the cross-shear zone 1 is formed in the R3 region. It can be seen that the second peak value (29.8 MPa) of Mg plate shear stress appears in this cross-shear zone 1 (Figure 7b). In the R4 region, the direction of the Mg shear stress changes again and the shear stress value fluctuated steadily. In the R5 region, the shear stress direction on the Al lower surface changes. The shear stress directions of the Mg upper surface and the Al lower surface are opposite, forming cross-shear zone 2 in the R5 region. The junction of the R6 region and R7 region is the peak position of the Mg plate, so it shows a sudden drop of shear stress in the curve.

The cross-shear zones formed in the corrugated rolling deformation zone have been marked with rectangles in Figure 7a,b. In addition to the ordinary compression deformation, the metals in the cross-shear zone are also acted on by the additional shear deformation [23]. The existence of the cross-shear zones will accelerate the fracture of the hard brittle layer and the exposure of the fresh metal, improve the interfacial bonding strength [24]. For the Mg/Al clad plate, there will be more slip systems of Mg plate in the cross-shear zone. The slip system type will also increase, and the deformation will be more severe. More sub-grains can be formed during severe plastic deformation, which can further promote the transformation of sub-grains to large-angle grain boundaries, thus completing the grain refinement process [25]. In addition, dynamic recrystallization is more likely to occur in Mg plate, which can also refine the grain and improve the Mg plate plastic deformation ability.

### 3.4. Tensile-Shear Property

**Figure 8:**
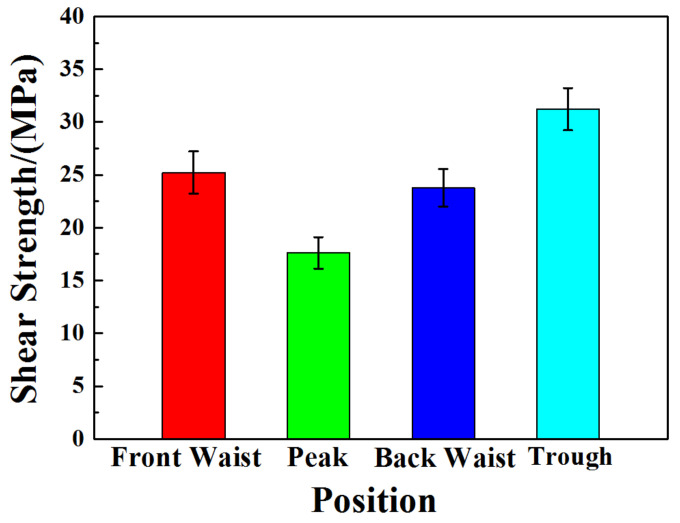
Transverse tensile-shear strength at four positions of the corrugated Mg/Al clad plate.

The statistical diagram of transverse tensile-shear strength at four positions of the corrugated Mg/Al clad plate is displayed in Figure 8. Tensile-shear strength can be calculated through τ_b_ = F/A [26], where τ_b_ is the strength, F is tensile-shear force, A is the bonding area. It can be seen from Figure 8 that the tensile-shear strength at the trough position is the largest, 31.22 MPa, but the tensile-shear strength at the peak position is 17.61 MPa. The tensile-shear strength at the front waist (25.21 MPa) and the back waist (23.77 MPa) is in the middle, and the strength at the front waist is slightly higher than that at the back waist. From the previous simulation results, the stress and strain of metals at four typical positions of the corrugated Mg/Al clad plate are different. The values of normal stress and normal strain on trough metals are the largest, while the values on peak metals are the smallest. The interfacial bonding strength of the clad plate is closely related to the fresh metal exposed on the metal surface to be bonded. The fresher the metal, the tighter the interfacial bonding strength. A large amount of fresh metal can be produced on the trough metal with large plastic deformation. These fresh metals will be squeeze into the other metal matrix to form a tightly bonded interface under the action of rolling force. However, the plastic deformation degree of peak metal is the smallest and a small amount of fresh metal is exposed, so the interface bonding strength at the peak position is the lowest.

**Figure 9:**
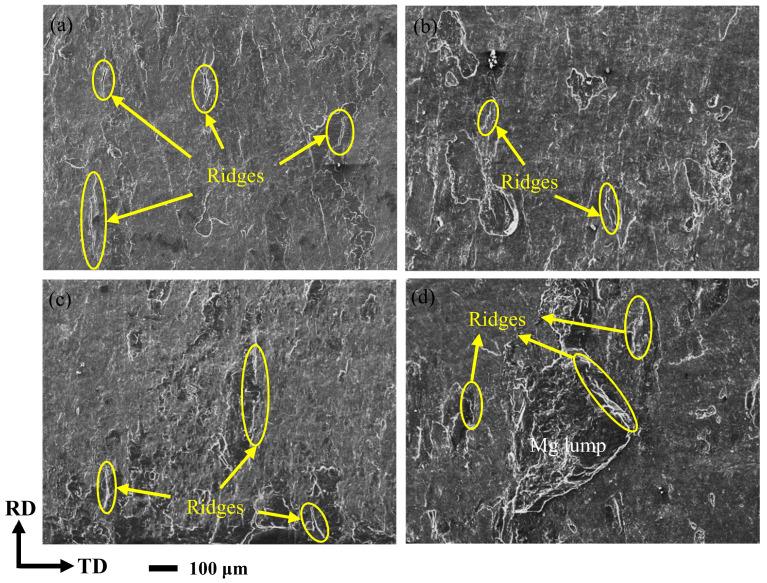
Tensile-shear fracture SEM images on the Al-side of the corrugated Mg/Al clad plate along the TD: (**a**) front waist, (**b**) peak, (**c**) back waist, (**d**) trough.

Figure 9 is the fracture SEM images at the Al matrix side of the corrugated Mg/Al clad plate after the tensile-shear test at four positions. It can be seen that the tearing ridges are contained on the Al matrix side at four positions, as shown in the yellow ellipses in Figure 9a–d. The metal Mg and metal Al are separated when the Mg/Al clad plate is pulled. Mg alloys are firmly adhered to the Al matrix because of the tight bonding interface, and the tearing ridges are formed after the complete separation [27]. At the peak position (Figure 9b), the tearing ridges are short and a few. At the trough position (Figure 9d), an adhesive Mg lump can also be found on the side of the Al matrix in addition to the tearing ridges. This is mainly due to the high reduction ratio at the trough position. The metals are broken under the action of high rolling force to form cracks, and the fresh metal is extruded from these cracks and squeezed into the opposite matrix, thus forming a tight bond interface [28,29]. The number of tearing ridges at the front waist (Figure 9a) and the back waist (Figure 9c) is between the peak position and trough position.

**Figure 10:**
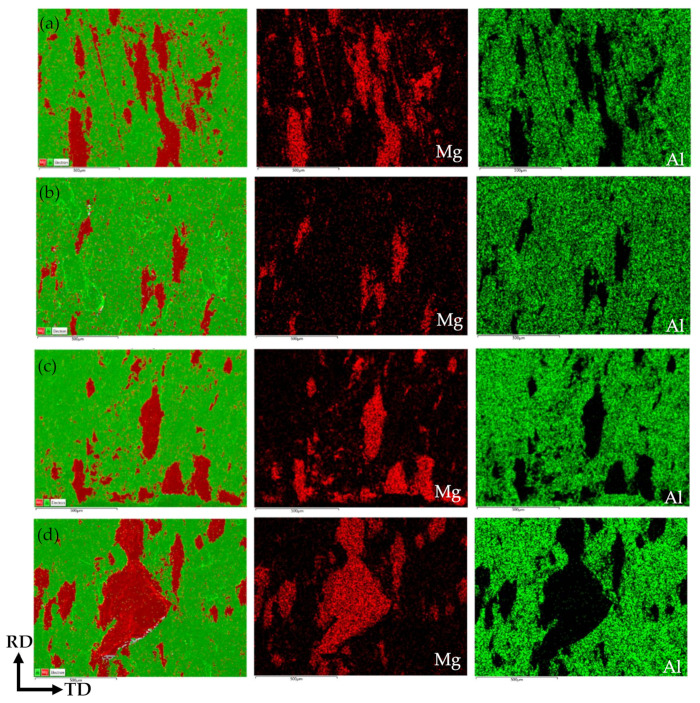
Element distribution of the transverse tensile-shear fractures on the Al-side of the corrugated Mg/Al clad plate: (**a**) front waist, (**b**) peak, (**c**) back waist, (**d**) trough.

Figure 10 shows the surface-scan distribution at the fracture surface of the corrugated Mg/Al clad plate after the tensile-shear test at four positions. In the surface-scan images, green represents the Al matrix metal, and red represents the metal Mg adhered to the Al matrix. The adhesion amount of metal Mg on the Al matrix at four positions is counted, and the statistical results are shown in Table 4. It can be seen from Table 4 that in the Al matrix, the position of Mg adhesion from more to less is trough-front waist-back waist-peak, which exactly corresponds to the transverse tensile-shear strength of the corrugated Mg/Al clad plate at four positions. This is largely due to the uneven stress distribution caused by the action of corrugated roller on the metals. It can also be seen from the scanning of tensile-shear fracture surface in Figure 10 that a large Mg lump adheres to the Al substrate at trough position (Figure 10d). At the front waist position (Figure 10a) and back waist position (Figure 10c), the metal Mg adheres to the Al matrix in a strip discontinuous distribution along the rolling direction. Although the adhesion of metal Mg can also be observed at the peak position (Figure 10b), the content is less, and the distribution is scattered and not concentrated. In addition, numerous dimples can be observed at the trough fracture surface after tearing of magnesium, which is also an important manifestation of the high bonding strength at the trough position.

## 4. Conclusions

Corrugated Mg/Al clad plate was successfully manufactured by an upper corrugated roller and a lower flat roller at 400 °C rolling temperature and 35% reduction ratio. The interface bonding performance of the corrugated plate was studied by the tensile-shear test. The finite element method was used to simulate the corrugated rolling process, and the interface bonding performance difference of the corrugated plate was explained by analyzing the stress and strain distribution in the rolling deformation zone. The main conclusions are as follows:

After the corrugated rolling, the surface of Mg plate presents an obvious corrugation shape, and four typical positions (front waist, peak, back waist, and trough) are defined according to the rolling direction and the corrugation morphology. The corrugated Mg/Al clad plate has a tight bonding interface, crack and intermetallic compound layer cannot be observed at the interface.

Corrugated Mg/Al clad plate has excellent interfacial bonding strength. The transverse tensile-shear strength at the trough position reaches 31.22 MPa, and the tensile-shear strength at the peak position is 17.61 MPa, and the front waist and the back waist are located in the middle.

It can be found that the stress and strain of interface metal at the trough position are the largest through numerical simulation results. Two cross-shear zones are formed in the rolling deformation zone of Mg/Al clad plate fabricated by corrugated roller, which can accelerate the metals plastic flow and promote interface close bonding.

## Figures and Tables

**Table 1:** Chemical compositions (in wt.%) of AZ31B Mg plate and 5052 Al plate.

Materials	Mg	Cu	Ca	Mn	Si	Al	Zn	Cr	Fe
Mg plate	Rest	0.01	0.04	0.8	0.07	3.2	1.2	-	-
Al plate	2.2–2.8	0.1	-	0.1	0.25	Rest	0.1	0.15–0.35	0.4

**Table 2:** Mechanical properties of AZ31B Mg plate and 5052 Al plate at room temperature.

Materials	Ultimate Tensile Strength (MPa)	Yield Strength (MPa)	Fracture Elongation (%)
Mg plate	284	171	24
Al plate	217	135	18

**Table 3:** Surface roughness of interface Mg plate and Al plate of the corrugated clad plate.

		Front Waist	Peak	Back Waist	Trough
	
Mg plate		3.205 μm	2.803 μm	3.121 μm	3.597 μm
Al plate		3.985 μm	3.157 μm	3.386 μm	4.430 μm

**Table 4:** The percentage of Mg element and Al element shown in Figure 10.

Map Images	Mg (%)	Al (%)
(a)-Front waist	22.46%	77.54%
(b)-Peak	8.29%	91.71%
(c)-Back waist	16.3%	83.7%
(d)-Trough	29.34%	70.66%

## Data Availability

Not applicable.
